# A Mixed Methods Study of the Challenges and Prospects of Utilizing Telemedicine in the Delivery of Healthcare to Nigerian Children

**DOI:** 10.3389/ijph.2025.1607790

**Published:** 2025-06-19

**Authors:** Igoche David Peter, Kuyet Jemimah Danjuma-Karau, Ejemeirele M. Omokhuale, Joel Cherima Yakubu

**Affiliations:** ^1^ Division of Paediatric Cardiology, Limi Children’s Hospital, Abuja, Nigeria; ^2^ Department of Anesthesia, Federal Medical Centre, Abuja, Nigeria; ^3^ John Snow International, Abuja, Nigeria

**Keywords:** telemedicine, SDG, healthcare delivery, newborn mortality, under 5th mortality

## Abstract

**Objectives:**

In Nigeria, telemedicine is underutilized despite unacceptable age-related childhood mortality indices. This study identifies the barriers, prospects, and benefits of telemedicine utilization in paediatric care in Nigeria.

**Methods:**

A convergent parallel approach of mixed methods design. Electronic questionnaires were used to obtain data from 57 and 50 mothers in an urban and a rural healthcare facility, respectively, in Abuja, Nigeria. Audio-recorded semi structured in-depth interviews were conducted with key informants, including a paediatrician, an ICT expert, and a matron. The qualitative data were analyzed via an inductive thematic analysis.

**Results:**

Telemedicine awareness was greater among urban respondents (p < 0.000). It was seen as inferior to physical consultation, and lack of awareness, cost of service, with resource constraints were barriers to utilization. Respondents unaware of telemedicine were 0.27 times less willing to pay for the services (p = 0.017). Themes generated include resource constraints, standard operating procedures, and possible advantages.

**Conclusion:**

Telemedicine is beneficial in terms of patient convenience and physical workspace decongestion. Public awareness and resource availability will enhance telemedicine utilization in paediatric care.

## Introduction

Telemedicine is defined as the practice of medicine via a remote electronic interface and is distinct from physical doctor-to-patient healthcare delivery [1]. Such interactions can occur using widely available smartphones or other mobile devices, two-way videos, etc. This technological tool has revolutionized healthcare delivery, as it provides the opportunity where without physically referring a sick child, doctors can request expert consultations via a live interactive synchronous audiovisual link from experts who are not physically present [2]. This has undoubtedly opened up access to quality healthcare for less privileged, improved family-centered care, reduced inconvenience and cost of patient transfers and the possibility of bridging the gap between the primary, secondary and tertiary levels of healthcare in an ICT-enabled healthcare ecosystem.

Although Nigeria is among the four countries (including Ethiopia, Ghana and Gambia) in sub-Saharan Africa that first established a telemedicine project in 1980, it has lagged in the application of telemedicine in child and maternal health, which Uganda and Kenya launched since 1982 even as Tanzania also established a tele-paediatric programme in 2008 [3]. The need for improved access to care among children aged less than 5 years is crucial considering that Nigeria, the most populous black country, accounts for an unacceptable 79% of the 2.4 million newborn deaths globally, and this is attributable to unfavourable social determinants of health [4]. Furthermore, relative to the developed world, the alarming 2021 underfive mortality and neonatal mortality rates of 110.8 and 34.9 per 1,000 live births, respectively, in Nigeria [5] motivated us to explore proven strategies that augment our existing physical doctor-to-patient consultations with alternatives such as telemedicine for healthcare in this age group, as this group has immense potential that is yet untapped.

Although it seems conceivable that telemedicine would be of immense benefit in the attainment of universal health coverage for Nigerian children with poorer chances of survival than should be, a scrutiny of the key players in the implementation of telemedicine revealed formidable obstacles such as a dearth of caregivers, unawareness and poor use buy-in, infrastructural deficits and technical issues [6, 7].

Most of the existing research has taken a quantitative approach, hence lacking the rich insight and texture that a qualitative approach could provide on telemedicine; hence, the present study intends to fill this methodological research gap by performing a mixed methods study. Furthermore, this study intends to fill a contextual research gap by identifying the barriers, prospects and anticipated benefits of the application of telemedicine in newborn and child health to attain the 2nd target of the 3rd Sustainable Development Goal.

## Methods

### Study Design

This mixed-methods study design used a convergent parallel approach, as it had to provide answers to different research questions that required different types of data and approaches. The quantitative arm used a cross-sectional approach, while the qualitative arm used a phenomenological approach. With the convergent parallel design, both quantitative and qualitative elements were conducted in the same phase of the research process, analyzed independently, and results interpreted together.

### Study Population

In the quantitative aspect of this study, respondents were selected from a list of mothers whose children were to receive immunization for the day from urban (Limi Children’s Hospital, Wuse 2, Abuja) and rural (Primary Healthcare Centre, Dawaki, Abuja) healthcare facilities in Abuja, FCT. The interviewers administered electronic questionnaires to obtain the data. Caregivers other than mothers were excluded.

In the qualitative arm of the research, key informants, including an ICT expert and a consultant paediatrician from both the urban health centre and the Chief Matron from the rural health centre, were interviewed.

### Determination of Sample Size

For the quantitative arm of the study, we used a telemedicine awareness level of 37.1% among respondents of a previous study by Arize and Onwujekwe in Enugu, Nigeria [8], and a population average of 60 mothers who were immunized for their babies per month at the urban study center, confidence limits of 5%, and confidence levels of 95%. A calculated sample size of 51 each from the urban and rural facilities was obtained from the Epi Info^®^ mobile app. Socioeconomic classification was performed using the revised scoring scheme proposed by Ibadin and Akpede [9].

### Data Analysis

The data obtained from the quantitative study are presented as percentages, tables and bar charts. Associations between nominal categorical variables were determined using chi-square tests, and predictions were made using binary logistic regression models in SPSS software^®^. Significant p value was set at < 0.05. The digital audio recordings, which were up to 20 min each from the in-depth interviews, were transcribed by Kaltura^®^ software, and the transcripts were validated against the material recorded by the interviewer. Two of the researchers generated codes from all the data, and any discrepancies were discussed until a consensus was reached. The coding system was refined until no further codes emerged. We conducted an open, inductive analysis, starting with open coding and following the steps for conventional content analysis [10].

### Triangulation

Data triangulation was performed as interviews were conducted on three different classes of key sources, including an ICT expert, a paediatrician and a matron. Additionally, methodological triangulation was achieved by the mixed methods design of this study.

## Results

One hundred and seven mothers (57 recruited from an urban healthcare facility and 50 from a rural facility) completed the questionnaire for the quantitative arm of this study, with the modal age category of 31–40 years and the least being ≤20 years, as shown in Table 1 below. Three key informants had in-depth interviews in the qualitative arm (Table 2).

**TABLE 1:** Age distribution of informants/mothers (Abuja, Nigeria. 2023).

Age category (years)	Frequency	%
≤20	1	0.9
21–30	37	34.6
31–40	53	49.5
41–50	13	12.1
>50	3	2.8
Total	107	100.0

**TABLE 2:** Characteristics of the key informants interviewed (Abuja, Nigeria. 2023).

Key informant	Gender	Years of experience	Practice location
ICT expert	Male	12	Urban
Consultant Paediatrician	Male	13	Urban
Chief Matron	Female	15	Rural

The respondents in the quantitative study arm were mostly in the middle socioeconomic class; 38 (35.5%) and 33 (30.8%) being urban and rural respondents respectively. 17 rural respondents (15.9%), and no urban respondents in the lower socioeconomic class. And 19 (17.8%) urban respondents and no rural respondents were from the upper socioeconomic class.

History of previous utilization of telemedicine was reported by only 18 (31.6%) of the urban area respondents and by none from the rural area.

There was a significant association between respondents’ location (urban/rural) and awareness of telemedicine consultation. Although more people were willing to pay than not to pay for both urban and rural respondents, this difference was not statistically significant (p = 0.079) (Table 3). Thirty-eight of the 41 urban respondents who had heard of telemedicine volunteered to explain their understanding of telemedicine, which included key words such as virtual consultation, online consultation, remote access consultation, and consultation using a mobile device.

**TABLE 3:** Telemedicine characteristics of 57 urban and 50 rural residents (Abuja, Nigeria. 2023).

Parameter	Urban n (%)	Rural n (%)	p- value
Ever heard of “telemedicine” Yes	41 (71.9)	5 (10)	0.001*
No	16 (28.1)	45 (90)	
Willing to pay for child’s telemedicine consultation			
Yes	48 (84.2)	35 (70)	0.079
No	9 (15.8)	15 (30)	

Keys: χ^2^ = Chi square, * = Statistically significant.

The reasons for 24 respondents’ unwillingness to pay for a telemedicine consultation included the following:

i. Perceptions.
  a. Telemedicine consultations lack the “real feeling” of doctor‒patient interaction (33.3%).
  b. Telemedicine consultation is not as in-depth as traditional physical consultation; 12.5%.
  c. Telemedicine consultation is deficient with regard to the physical examination aspect of consultation (20.8%).
ii. Lack of awareness.
  a. Telemedicine consultation was an unknown concept to me (16.7%).
iii. Dual costing: for data and actual consultation
  a. Telemedicine consultation already costs my money on data; 16.7%.

The modal consultation fee acceptable for telemedicine consultation by all respondents was 5000 naira, and acceptance decreased with increasing consultation fees.

Regarding teledensity, all the urban area respondents own a smartphone and can access internet services on their mobile devices, while in the rural area, 45 (90%) and 42 (84%) respondents own a smartphone and access the internet, respectively. Among the urban respondents, 82.5% had better smartphone internet proficiency, 14.0% had excellent proficiency, 2.0% had fair proficiency, 38.0% had excellent proficiency, 32.0% had good proficiency, 12.0% had fair proficiency, and 16.0% had poor proficiency in rural areas. The availability of mobile network services was poorer in the rural areas, with 10.0% having no such services, 28.0% fluctuating and 62.0% having good mobile network services, compared with 96.5% of the urban respondents who had good network services, with only 3.5% having fluctuating networks. The electricity supply was better for urban respondents than for rural respondents.

**FIGURE 1:**
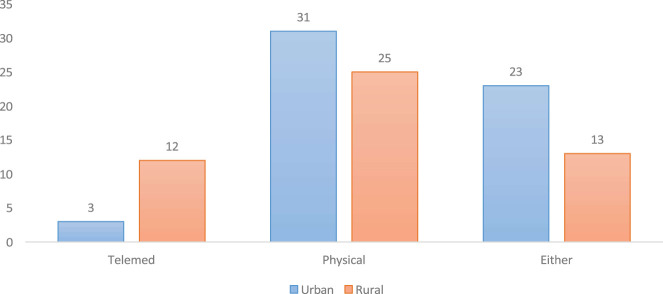
Preference for consultation modes among urban and rural respondents (Abuja, Nigeria. 2023).

Interestingly, more rural centre respondents preferred telemedicine than did urban respondents, although the majority of all respondents preferred physical consultation. The choice of either telemedicine or physical consultation was greater than that of telemedicine (Figure 1), and the reasons given for the choice of preference included illness severity (55.6%), convenience (40.4%), and network availability (4.0%, all from the rural respondents).

Concerning predictors of “willingness to pay” for telemedicine consultation for their children, mothers who had never heard of telemedicine consultation were 0.27 times less likely to pay than those who had ever heard of it (p = 0.017) (Table 4). Surprisingly, neither socioeconomic class nor the amount of consultation fees were suitable predictor variables for willingness to pay for telemedicine consultation according to binary logistic regression models.

**TABLE 4:** Adjusted odds ratios with 95% confidence intervals for binary logistic regression models used to determine predictors of willingness to pay for telemedicine consultation (Abuja, Nigeria. 2023).

	Adjusted odds ratio (95% p- value confidence interval)	
Ever heard of telemedicine consultation		
Yes	1.00	
No	0.27 (0.092–0.790)	0.017

On thematic analysis, we found three main themes divided into subthemes (Table 5).

**TABLE 5:** Themes and subthemes related to the use of telemedicine (Abuja, Nigeria. 2023).

Resource constraints	Standard operating procedures	Possible advantages
*Infrastructure	*Case selection guidelines	*Decongestion of physical workspace
*Manpower	*Quality assurance	
*Affordability		*Convenience

Theme 1 Resource constraints

### Infrastructure

All participants expressed concerns, albeit from different perspectives, about resource constraints that could hamper the smooth setup and operation of paediatric telemedicine consultations. This feeling stems from the physical, human and fiscal challenges common in developing country contexts.

From the ICT viewpoint, the infrastructure necessary for telemedicine consultations includes computers and/or smartphones/mobile devices with sufficiently well-defined cameras, software with a user-friendly graphical interface, and available internet services with sufficient bandwidth. The ICT expert noted that (I) 1 “equipment matter and how good the internet connectivity is”. Unfortunately, the dearth of suitable infrastructure was a recurring theme among the participants with Matron (M) 1, who lamented that “we do not have IT facilities nor experts to formally render telemedicine services. Additionally, when designing telemedicine software, options for language translations suitable for patients should be considered so that those who cannot speak English may still benefit”. As important as this logistics are, they are not readily available, and worse, they still vary across the socioeconomic divide.

### Manpower

Paediatricians and Matrons believe that even if the ICT infrastructure deficit is addressed, there is a dearth of trained personnel to provide medical care to sick children, especially with the current massive brain drain. This shortfall in manpower can, however, be reinforced, according to the Paediatrician who noted that (P) 1, “the few workforces is in difficult situation, but they may be guided by experts in another geographical location via the telemedicine platform”. (M) 2 also said,“Telemedicine will help augment the clinical service providers’ workforce during shift duties with low staff strength.”

### Affordability

The interviewees are concerned about the affordability of consultation fees for telemedicine because the end user is charged both for data usage and for actual consultation, which may discourage patronage in our environment. According to (I) 2, “in more developed climes, some services are free on the internet, so if you want to access telemedicine for instance, it does not charge you from your mobile plan, and this can help encourage user buy-in.” For telemedicine services to be patronized, (M) 3 “mothers should be empowered.”

Theme 2 Standard operating procedures

### Patient Selection Guidelines

As promising as telemedicine presents, it was interesting to realize that not all medical conditions in newborns and children aged less than 5 years can be consulted virtually; hence, selection criteria for cases that are suitable for telemedicine should be established. Participants reported that cases for virtual consultations should be selected on the basis of illness severity and the extent of need for physical examination details. (P)2: “Things that increase parental anxiety, which may not be life threatening, such as a newborn being overcloothed and baby having high temperature, a telemedicine consultation can take care of that, as they are reassured. Telemedicine should be restricted to medical conditions that are mild.” However, moderate to severe medical conditions can also be handled on the basis that medical personnel with some basic skill set are physically present with the patient and are videoconferencing with another expert for collaborative practice.

### Quality Assurance

Since the healthcare provider must not be in the formal hospital setting to render services, it is thought that to maintain the standards of clinical care, teleconsultations should be monitored closely. (P) 3; “there is need to ensure quality control and censor services rendered so that errors are rectified and quick follow-up actions taken.” It is hoped that once the end-users are sure that the minimum standards of clinical consultations are met with telemedicine, they will have easy access to quality healthcare and will be less likely to patronize unsolicited wrong or incomplete advice, which is prevalent in our environment.

Theme 3 Possible advantages

### Decongestion of the Physical Workspace

Instead of having all cases present physically to the hospital with resultant overcrowding and risk of spread of some communicable diseases, the participants believe that consultations performed for selected cases by videoconferencing will obviate this challenge. They noted that clinicians will then have a conducive atmosphere to focus on those cases that must present physically.

### Convenience

The fact that a qualified healthcare provider can be consulted from the comfort of home is a major attractive feature of telemedicine. One participant explained (M): “At least you save the money and stress of transportation.”

## Discussion

Our findings present a significant dissimilarity between the cohorts of our urban and rural respondents in terms of awareness of telemedicine (p < 0.000), which is conceivable from the divide in terms of socioeconomic status, as none of our urban respondents belong to the lower socioeconomic class and *vice versa*. Although Arize and Onwujekwe in Enugu, Nigeria, found that the aggregate majority of their respondents at the heterogeneous study population level were unaware of telemedicine, they reported findings similar to ours when they dichotomized the respondents with respect to socioeconomic class. Interestingly, Ajala et al. [11] reported an appreciable 85% increase in telemedicine awareness in southwestern Nigeria, but their study population differed from ours since they studied healthcare providers against the service consumers who were our respondents. Although their reported awareness seems impressive from a cursory glance, we are baffled that not all healthcare providers who are literate are even aware of telemedicine. If this is the case in their study location, then it is conceivable that the supposed end users may be bereft of this knowledge, as one of our interviewees explained. Telemedicine itself is a foreign concept, so if there’s a way that we can localize it, so that people understand it; if I say tele-medicine, most people will be clueless about it. However, if an individual says an online consultation with a doctor via phone or a phone consultation, it may be more acceptable. Among both the urban and rural cohorts of respondents, those willing to pay for telemedicine services were more likely than those unwilling to, although this difference was not statistically significant (p = 0.079). We found a much greater “willingness to pay” for telemedicine consultation (84.2% and 70% for urban and rural respondents, respectively) than the 48.7% reported by Arize and Onwujekwe [8] and the mere 25% among older Japanese patients during the COVID-19 pandemic [12]. This contrasting observation may not be connected with the level of respondents’ awareness between our study and that of the Enugu’s and the age disparity between our respondents and the Japanese people who were aged above 70 years, as we assume that older people are less tech-savvy.

The present study predicted that respondents who were unaware of telemedicine would be less likely to pay (p = 0.017) than those who had ever heard of it. Hence, awareness was a significant predictor of a buy-in to telemedicine. In contrast to our observation, socioeconomic status was a predictor in the study by Arize and Onwujekwe [8]. This disparity may be attributable to the fact that the schemes used for socioeconomic status differ; we used Ibadin and Akpede [9], who published a revised standardized scoring scheme for the classification of socioeconomic status, whereas the former determined their own method.

Although we had more respondents who were willing to pay for telemedicine, we explored the reasons for this, including awareness, perceptions, and fees. We found that 16.75% of the respondents were unwilling to pay because they were unaware of telemedicine and were uncomfortable with the dual data/consultation payments for telemedicine. To address these obstacles to telemedicine utilization in child healthcare, an interviewee suggested that the public should be informed about the option of telemedicine and that women should be empowered. However, other respondents believed that telemedicine lacked the “real feeling” of doctor‒patient interaction (33.3%), was not “in-depth” (12.5%), and was deficient with regard to physical examination (20.8%). This observation is similar to that of Maria et al. [6] in Portugal, who reported that patients believe that face-to-face consultations are more valuable because they provide room for better communication and relational closeness.

Although teledensity was excellent among our study respondents, barriers militating against telemedicine services, apart from the aforementioned poor awareness, included poorer technological literacy, poorer mobile network services and poorer electricity supply for the rural respondents, and similar observations were made by Galle et al. [7]. In a rural health center, the interviewee revealed that some of the clients here did not even have mobile phones until now. When asked of their phone numbers, they do not have. These patients cannot benefit from telemedicine services. It is obvious that disadvantaged rural dwellers have worse social determinants of health. This means there is more to be done in terms of infrastructure and social support to bridge the gap between urban and rural areas. Considering that all our interviewees further emphasized the invaluable position of resources necessary for implementing functional telemedicine services and their unfortunate dearth in our environment, it is obvious that establishing telemedicine is still a far cry here.

Notwithstanding the seemingly daunting challenges facing the implementation of functional telemedicine services, it is pertinent to highlight its benefits as applicable to newborn and child health and hence contribute to the prevention of avoidable mortalities. Access to quality newborn care will be enhanced as subspecialist support from existing healthcare providers becomes feasible, obviating geographical constraints, in tandem with the observations of Azzuqu et al. [13]. Furthermore, for selected cases, as reiterated by our interviewees, these newborns need not leave the comfort of their homes for a telemedicine consultation; hence, they receive the advantage of family-centered care, which is also more affordable than what is obtained in physical consultations, thus agreeing with other scholars [14, 15]. Furthermore, on the telemedicine platform, older children also have the golden opportunity to benefit from remote consultations with subspecialists and options for the review of paediatric ECGs, including advanced technology for tele- auscultations using a digital stethoscope and EEGs by paediatric cardiologists and paediatric neurologists, respectively.

### Conclusion

While telemedicine service providers will leverage on availability of stable telecommunications network services in both rural and urban areas, the end-users will need awareness campaigns, adequate electric power supply, and the healthcare organizations need initiatives to ensure confidentiality of medical records. Although telemedicine is useful for newborn and child health, unambiguous case-selection criteria with clear management guidelines are necessary.

### Recommendation

As a means of contributing to the care of newborns and children aged less than 5 years, telemedicine should augment physical doctor-to-patient consultation, especially when a specialist opinion is needed. Attention should be given to awareness of telemedicine as an option for conventional physical consultation. The technological infrastructure and financial empowerment of end-users necessary for the actualization of telemedicine should receive more attention, especially in rural areas.

### Limitations

The quantitative arm of this study is limited to respondents drawn from mothers visiting healthcare facilities, with slightly more urban respondents than rural. The inclusion of respondents at the general population level would provide a deeper understanding of the concept studied. Although three different categories of interviewees were studied, further studies could consider interviewing more subjects per category until data saturation is attained.

## Data Availability

The data that support the findings of this study are available from IP, but restrictions apply to the availability of these data, which were used under licence for the current study and so are not publicly available. The data are, however, available from the authors upon reasonable request and with permission from IP.
